# A Retrospective Study of the Effects of COVID-19 Non-Pharmaceutical Interventions on Influenza in Canada

**DOI:** 10.3390/idr17030059

**Published:** 2025-05-26

**Authors:** Heather MacTavish, Kenzie MacIntyre, Paniz Zadeh, Matthew Betti

**Affiliations:** 1 Department of Biology, Mount Allison University, Sackville, NB E4L 1E2, Canada; 2 Department of Math & Computer Science, Mount Allison University, Sackville, NB E4L 1E2, Canada

**Keywords:** influenza, COVID-19, non-pharmaceutical interventions

## Abstract

**Background/Objectives**: COVID-19 pandemic had a significant impact on endemic respiratory illnesses. Through behavioral changes in populations and government policy, mainly through non-pharmaceutical interventions (NPIs), Canada saw historic lows in the number of influenza A cases from 2020 through 2022. In this study, we use historical influenza A data for Canada and three provincial jurisdictions within Canada—Ontario, Quebec, and Alberta—to quantify the effects of these NPIs on influenza A. **Methods**: We aim to see which base parameters and derived parameters of an SIR model are most affected by NPIs. We fit a simple SIR model to historical influenza data to get average paramters for seasonal influenza. We then compare these parameters to those predicted by fitting influenza cases during the COVID-19 pandemic. **Results**: We find substantial differences in the effective population size and basic reproduction number during the COVID-19 pandemic. We also see the effects of fatigue and relaxation of NPIs when comparing the years 2020, 2021, and 2022. **Conclusions**: We find that the effective population size is the main driver of change to disease spread and discuss how these retrospective estimates can be used for future forecasting.

## 1. Introduction

The COVID-19 pandemic had a substantial impact on every facet of daily life around the world. In Canada, the non-pharmaceutical interventions (NPIs) put in place for much of the time period beginning around March 2020 to approximately the end of 2022 had a profound impact on other endemic respiratory infections.

Beginning in March 2020, jurisdictions across Canada experienced a variety of measures that could and did impact the spread of airborne infectious diseases [1,2]. These measures included lockdowns [3,4], masking (both on a personal and institutional level [5,6]), and social distancing [7]. While the adherence, enforcement, and austerity of these measures in Canada differed by province [8], they left a measurable effect on seasonal influenza.

Almost immediately, as NPIs took hold, a measurable effect was seen on influenza testing as reported by FluWatch [9]. In fact, it is possible that one of the two major lineages of influenza B may have been driven to extinction through COVID-19 and associated NPIs [10].

Since Canada has ‘returned to normal’, there have been studies that have focused on the interplay between vaccination, non-pharmaceutical interventions and policies, and the differing outcomes of jurisdictions as a result [11].

Influenza has long and thoroughly been studied using mathematical modeling. Using compartmental models to study the spread of infectious diseases has been in practice for over a century, with work being conducted as early as 1911 [12]. The compartmental SIR framework, where a population is broken into three classes— (S)usceptible, (I)nfectious, and (R)ecovered—is a popular choice for influenza modeling [13] and can be easily adapted to study different aspects of an epidemic such as cross-immunity from previous infections [14], or vaccination strategies [15]. The SIR model is often well suited for parameter estimation of key epidemiological parameters, given appropriate population-level data [16].

Unfortunately, testing and reporting is often biased toward severe cases [17], and therefore will severely underestimate the true number of cases within a population. Since 2020, the amount of data collected—particularly for SARS-CoV-2 infections—has improved, leading to better estimates of parameters [18]. Meanwhile, at least in Canada, the collection and reporting of influenza has not changed. Influenza tests are reported mainly through emergency departments and hospitalized severe acute respiratory infections [19]. Using archived records from pre-COVID (2019), we can see that the methods for laboratory-confirmed influenza A has not changed significantly from pre-pandemic methods [20]. While this can provide overestimates or underestimates of model parameters [21], in this study, we hope to circumvent the limits of available data by focusing on relative comparisons of parameters. This is further complicated at a national level, as different provinces and territories have different strategies for testing and reporting influenza [22].

The purpose of this study is to estimate and report relative changes in basic epidemiological parameters of influenza A in Canada due to the NPIs associated with COVID-19. Our aim is to provide insight into which parameters are most affected by changes in policies and behaviors, and that this might provide guidance on how to model a changing behavioral landscape in future pandemics.

Here, we use a simple SIR model coupled with influenza A data from Summer 1999 to Spring 2023 to estimate relative changes in the reproduction number, attack rate, effective susceptible population, and contact rate induced by NPIs and COVID-19 policy. An SIR model is chosen for its simplicity and to reduce the number of parameters that are fit. As influenza data are reported weekly, there are often approximately 50 data points per season. We find that, with the data available, NPIs have a much greater effect on the attack rate of an outbreak than the reproduction number. We discuss the interpretation and impacts of this sensitivity in the context of other endemic or emerging infectious diseases, and in relation to policy.

## 2. Methodology

### 2.1. Model

In this study, in order to reduce the effects of correlations, we use a simple SIR model with our historical flu data. This technique is not new, and is the basis for the forecasting of seasonal influenza in some studies [21,23], and is used to simulate data in other cases [24]. In implementing this model, we reduce the number of parameters that require fitting by fixing those that are less likely to change over the period of time we are considering (i.e., the rate of recovery). The effective starting susceptible population is highly variable year to year, as it depends on vaccination rates, vaccine efficacy, social behaviors, weather, etc. We fit this parameter and show that NPIs largely affect the effective population size.

Our model framework consists of a system of ordinary differential equations,

(1) $$\frac{dS}{dt}=-\beta SI$$

(2) $$\frac{dI}{dt}=\beta SI-\mu I$$

(3) $$\frac{dR}{dt}=\mu I$$

(4) $$\frac{dC}{dt}=\beta SI$$

(5) $$S(0)=S_{0}$$

(6) $$I(0)=I_{0}$$

(7) R(0)=0,

where *S* is the susceptible population, *I* is the infected population, and *R* is those that have recovered with immunity. The model is augmented with *C*, the cumulative case counts for the flu year. We do this so that we can fit both dC/dt (new cases per week) and C(t) (cumulative cases) to the data.

While $S_{0}$ is often taken to be the whole population, this is complicated for influenza due to potential cross-immunity [14] and vaccination [25]. We take $S_{0}$ then to be the effective susceptible population size at the beginning of the flu year. In the context of our study, this is further complicated as NPIs effectively remove individuals from the population in some capacity. Thus, we consider $S_{0}$ to be an *effective* initial susceptible population.

We likewise assume that the demographic changes over one flu year are negligible. These assumptions are again made to reduce the number of parameters of the model (to reduce covariance) and to keep the model mathematically tractable.

We estimate a seasonal reproduction number for each flu year using the standard expression for the reproduction number of an SIR model:

(8) $$\begin{array}{c} R_{S}=\frac{\beta }{\mu }S_{0} \end{array}$$

We do not start each year with a fully susceptible population, and thus, this is not a true basic reproduction number [26]. Our interpretation of $S_{0}$ as an effective population size leads to the interpretation of $R_{S}$ as a seasonal reproduction number. In other words, $R_{S}$ measures the effective reproduction of influenza A at the start of a season, and $S_{0}$ measures the number of people susceptible at the beginning of a flu year, thus discounting those with previous immunity, or those in isolation.

There are generally issues of identifiability between β and $S_{0}$. As we have full epidemic curves for each season, we are able to estimate both parameters. The estimate for β is largely driven by early epidemic dynamics, and $S_{0}$ is largely determined by late epidemic dynamics, through the estimate of the final size of the outbreak:

(9) Rinf=S01−e−R0−βμSinf.

By using this expression in fitting our data, we have $S_{0}$ decoupled from β, reducing the issues of parameter identifiability.

We use provincial and national influenza data from Canada obtained from the publicly available FluWatch [19]. Our interest is mainly in how model parameters differ from historical data in the 2020–2021 and 2021–2022 flu years, and which model parameters are most affected by societal changes induced by COVID-19.

**Figure 1:**
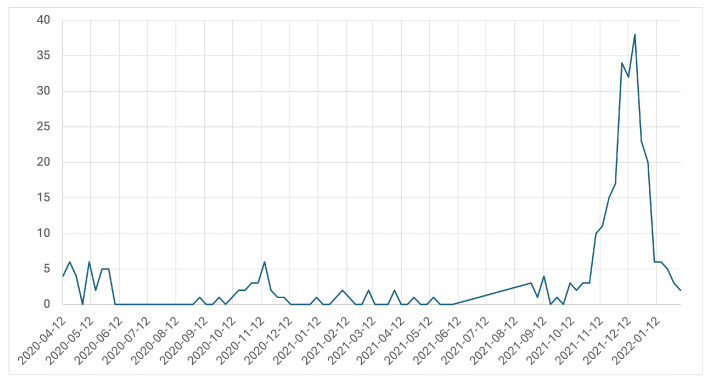
National influenza A incidence as reported in Canada. We note that, unlike with COVID-19 incidence during this time, we do not see the effects of provincially mandated lockdowns on influenza A. This leads us to treat NPIs as overarching behavioral and societal changes during this time, namely, social distancing and masking.

We can see in Figure 1 that behavioral changes induced by NPIs largely impact the incidence of influenza A uniformly. As such, when considering the impact of NPIs on the spread of influenza A, we discuss the large societal and behavioral changes present during the period of March 2020–March 2022. The sporadic lockdowns [27] that occurred throughout this period are not seen within these data, as they are within COVID-19 data (as illustrated in [27]). Thus, in this study, the NPIs mostly being considered are those at a societal and behavioral level such as social distancing and masking.

Influenza A vaccination data were only collected biennially prior to 2015 [28], and their efficacy on a per-year basis is difficult to estimate. We present vaccination data at a national level in Table 1 as gathered from Government of Canada sources [28,29,30]. At the provincial level, data are difficult to source and far more sparse. We can see in Table 1 that the rates of influenza vaccination across the country are relatively stable between 30% and 40%, with COVID-19 apparently influencing more individuals to obtain a vaccination against influenza A. Since it is fairly constant, we assume that the effects of vaccination fall into the uncaptured variance from year to year within our data and model.

### 2.2. Fitting

We fit the parameters of this model using least squares to minimize the error between the cumulative case counts per week and the new cases per week. We use both data sets, as, often, corrections and delays in data collection and reporting will be reflected in cumulative case counts but not in the weekly new case counts. By using both, we are able to minimize the effects of errors in data collection/reporting.

As the number of tests performed and number of cases observed vary between seasons, we measure the goodness of fit with the relative root mean square error,

(10) $$\begin{array}{c} RRMSE=\sqrt{\frac{1}{(N-P)m}\sum_{i=1}^{N}{\left(y_{i}-{\hat{y}}_{i}\right)}^{2}}, \end{array}$$

where *N* is the number of data points, *P* is the number of parameters fit, *m* is the mean of squares of all values fit, $y_{i}$ are the data points, and ${\hat{y}}_{i}$ are the fitted values. We use the relative root mean square error of both cumulative cases and daily incidence to give equal weight to the early epidemic, late epidemic, and the peak incidence.

By using both cumulative cases, new reported cases, and the final size estimate, we also are able to take into consideration the different scales of reported cases across an outbreak. If we only use cumulative case data for instance, our fitting would favor the later points in an outbreak, as the magnitude of these points will create a larger difference in the residual. We show in Figure 4 that this method does indeed produce a small relative error as well, indicating good fits to the beginning and end of an outbreak. This fitting method is used with great success for fitting COVID-19 outbreaks and predicting relevant epidemiological parameters in the face of NPIs [8].

Table 2 shows the adding of more complex dynamics to the model through a delay in infectiousness (commonly referred to as an SEIR model [31]). We see that the Akaike information criterion (AIC) [32] for the SIR model is lower on average than that of the SEIR model. This suggests that the data support the simpler model of the two models. Using this rationale, we can preclude more complex dynamics, as the data are unlikely to support such models without overfitting.

We compare two fitting algorithms per season—least squares and dual annealing—and take the better fit of the two in a relative root mean square error sense. We use least squares (gradient descent) for its speed and reliability, but given the broad sample space of parameters, the probabilistic dual-annealing algorithm increases the chances of finding a global minimum in the RRMSE [33] as opposed to a local minimum.

We fix μ=72 based on established data that the flu lasts approximately 2.5 days [25,34,35]. We do this, as we do not expect non-pharmaceutical interventions to change the rate of recovery of individuals within the population.

An influenza year is defined approximately from August of a given calendar year until July of the following calendar year. As an example, the 99-00 season runs from August 1999 through July 2000. The corresponding flu seasons for an influenza year run, typically, from November to March.

Seasons are fitted sequentially against data from Canada’s FluWatch [19] where available. We are able to find data for all years from 1999 to 2023, excluding 2010–2015. For Ontario and Quebec, provincial level databases, refs. [36,37], respectively, are used when FluWatch data was missing. By doing so, we are able to fill in missing FluWatch years at the provincial level. We use direct reported case values for cumulative cases and new cases as opposed to percent positivity.

Literature estimates are used for the initial guess at β [25]. We start from this particular estimate, as the data sets and model used to determine β in [25] are similar to our methods, and as we would like to use a consistent initial guess for each season, we take an estimate from approximately the middle of our data set, 2010. In [25], the authors similarly use historic influenza data with an SEIR framework to estimate a β value for Canada. While they ultimately focus on optimal vaccination strategies for pandemic influenza, their estimates of β are a valuable starting point for our own analysis due to the similar modeling framework being used over the same geographic region.

The initial susceptible population, $S_{0}$, is fit. We use the total number of tests performed in a season as a starting point for fitting; where this is unavailable, we use an estimated attack rate of 0.25 [25] and start fitting from the cumulative cases at the end of the season over the assumed attack rate.

Starting values and fixed values for parameter fitting are given in Table 3.

## 3. Results

**Figure 2:**
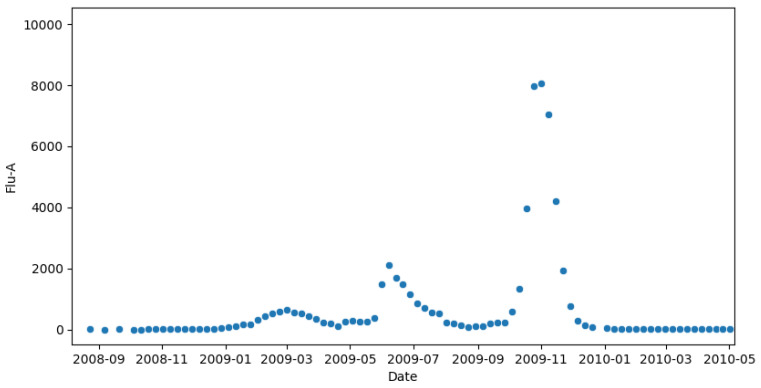
Influenza A data for 2009–2010. We see multiple peaks due to the concurrency of the H1N1 influenza pandemic and the regular seasonal influenza. Our model assumes a single pathogen with a single-peak outbreak and thus is incompatible with this year.

**Figure 3:**
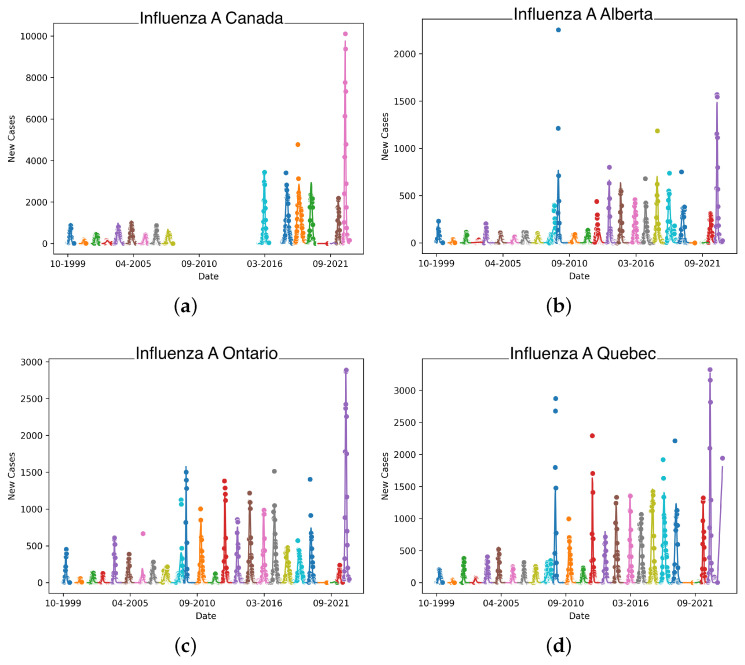
Influenza A data for Canada, Alberta, Ontario, and Quebec plotted per season starting from 1990 through 2023. The data are fit individually per season, as shown by the color variation. Gaps show missing seasons from the data set. Colours represent individual seasons.

The model is limited to single-pathogen outbreaks with a single peak. While this is true of seasonal influenza, Canada was heavily affected by the 2009 H1N1 pandemic [38]. As this was an influenza A pandemic, this resulted in the influenza A data in 2009 to reflect two pathogens. On a national level, this led to multiple peaks as seen in Figure 2. This is fundamentally incompatible with our model that leads to non-convergence and meaningless estimates. For this reason, the 2008–2009 and 2009–2010 years are excluded. In Figure 3, there is a gap in panel (a) which reflects the omission of the H1N1 pandemic and the years of missing data.

We see in the provincial panels of Figure 3 that the effects of H1N1 are less pronounced, allowing us to fit the data at this level. This is mainly due to the fact that the H1N1 cases are geographically “spread out” across all ten provinces, meaning we see its affect mostly in national-level data.

**Figure 4:**
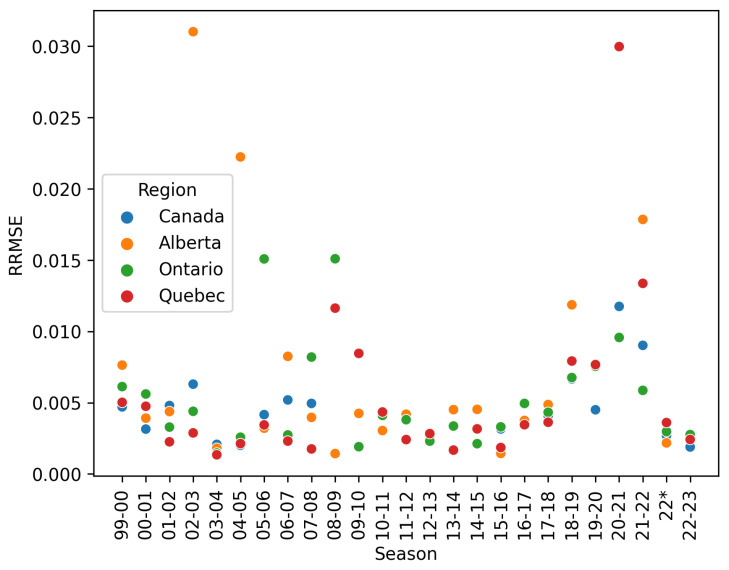
Root mean square error, Equation (10) for the best fit model for each season. We use the RRMSE and a visual inspection to flag and remove bad or non-convergent fits. Season 22 * indicates the secondary influenza season observed in 2022, which began in March and continued through the summer of 2022, as opposed to a typical influenza season which begins in November and ends in March.

Figure 4 shows the relative-root-mean-square error of the fit for each season, which remain generally <1%. While there are outliers, many of the worst fitting models can be explained by confounding factors, such as the introduction of H1N1 in 2009, straddling the 2008–2009 and 2009–2010 seasons, and the years 2020 through 2022 when NPIs significantly impacted the spread of influenza A in Canada.

Figure 3 shows these fits overlaid on the data for each season for across the four jurisdictions studied. Due to each season being considered in isolation, there is little continuity between the seasons. We see that the fitted curves generally agree with the data on a per-season basis.

Table 4 shows the estimates for β, $S_{0}$, $R_{S}$ and the attack rate for each flu year for Canada, Ontario, Alberta, and Quebec, for which at least one fitting method is converged. We note that Canada’s FluWatch does not report provincial-level data for the 2020–2021 season, or for proceeding seasons. For these seasons, provincial level data are used when available [36,37,39], As such, this season is absent from analysis. Other missing rows of data are indicative of non-convergent fitting or large (>2%) RRMSE.

Of particular interest are seasons 2020–2021 and 2021–2022, during which NPIs for COVID-19 were highly used, including increased masking by the general public, widespread social distancing, and intermittent lockdowns. We see that the effect is largely seen in the base parameters through effective susceptible population size, $S_{0}$, which translates to decreases in $R_{S}$ and attack rate. Of interest is that the derived parameters $R_{S}$ and attack rate in 2021–2022 seem to be much closer to pre-pandemic estimates; the effective susceptible population size for this season, shown in Table 4, is still well below historic levels.

**Figure 5:**
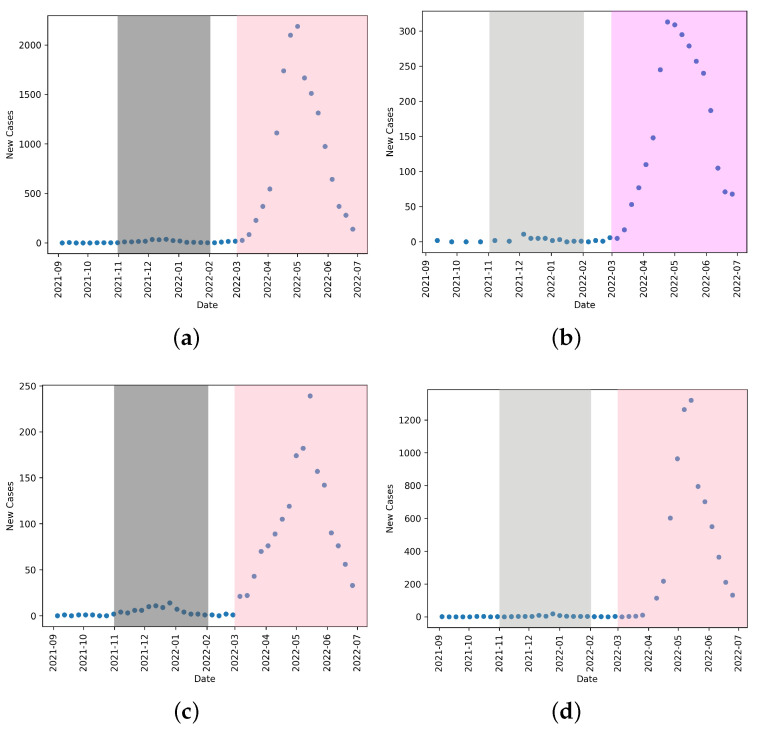
Influenza A reported cases for (**a**) Canada, (**b**) Alberta, (**c**) Ontario, and (**d**) Quebec. The grey shaded area shows the typical influenza A season, and the pink shaded area shows the shifted outbreak in 2022. This is common across all three provinces and nationally.

**Figure 6:**
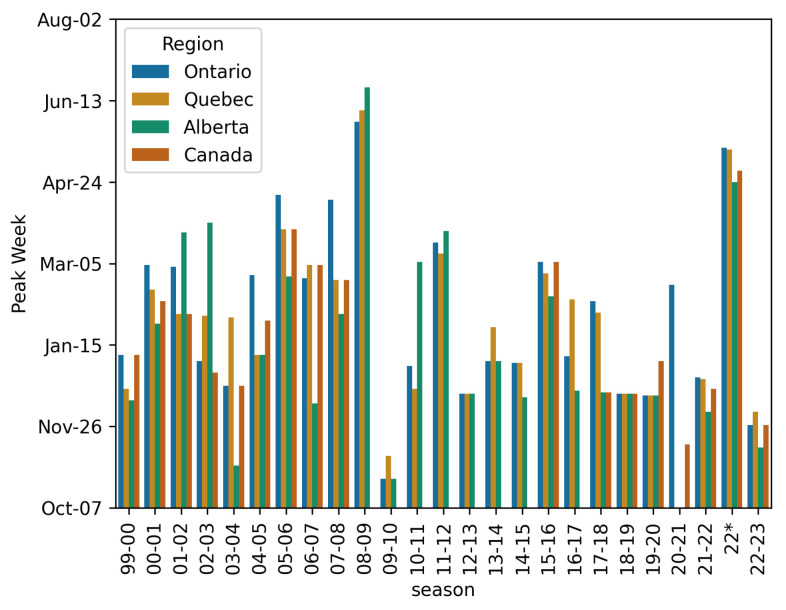
Plot showing the week with the most new influenza A reports per season for each of the four studied jurisdictions. We see that in 2022, there was an abnormal outbreak of influenza A in the spring. The year 08-09 is likely affected by the H1N1 pandemic which was officially declared in August 2009, straddling two influenza seasons.

We mark Spring/Summer 2022 as its own season, labeled in the figures as $22^{*}$ as the changes in NPIs created conditions for a secondary/delayed flu season in 2022. This can be seen in Figure 5 and Figure 6. We note that this uncharacteristic influenza A outbreak occurs across all four jurisdictions.

**Figure 7:**
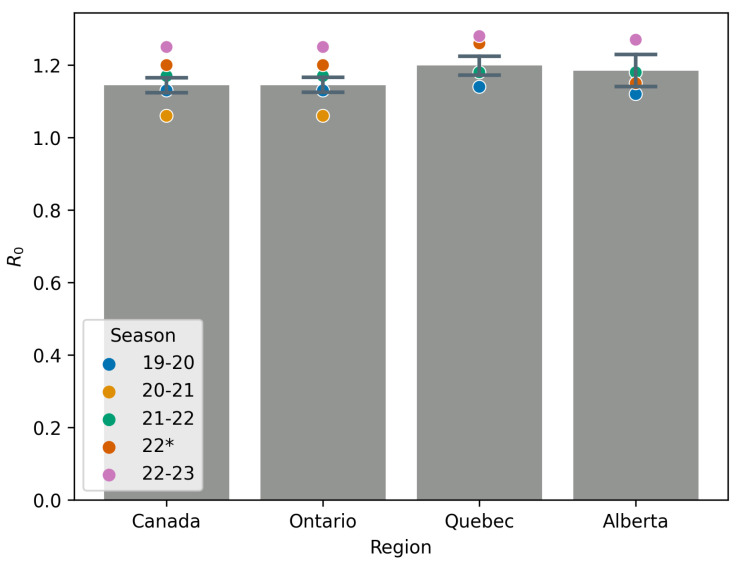
Mean $R_{S}$ values (height of grey bars) for all seasons pre-COVID (i.e., seasons 99–00 through 18–19); the season 09–10 is excluded from any computation due to the H1N1 pandemic. Point estimates are given for the 19–20, 20–21, 21–22, 22, and 22–23 seasons for comparison. Seasons 22* is a secondary outbreak of influenza caused by relaxation of NPIs in spring 2022.

**Figure 8:**
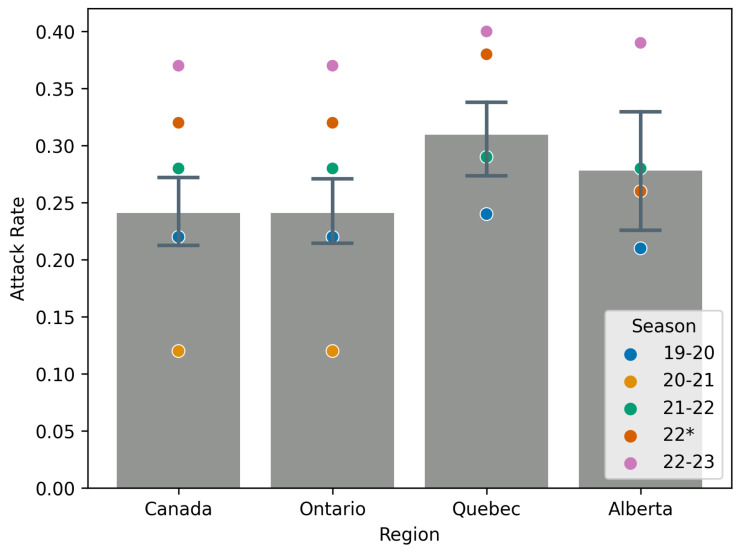
Mean attack rate values for all seasons pre-COVID (i.e., seasons 99–00 through 18–19). The season 09–10 is excluded from any computation due to the H1N1 pandemic. Point estimates are given for the 19–20, 20–21, 21–22, 22, and 22–23 seasons for comparison. Seasons 22* is a secondary outbreak of influenza caused by relaxation of NPIs in spring 2022.

Figure 7 shows the mean $R_{S}$ and 95% confidence interval for this mean for season 99-00 through 18-19, deemed pre-COVID, and how the seasons 19-20, 20-21, 21-22, 22, and 22-23 compare. Similarly, Figure 8 shows the mean attack rate and a 95% confidence interval for this mean for the seasons 99-00 through 18-19. Also on the figure are point estimates of the attack rate for the seasons 19-20 through 22-23. All mean values exclude the 09-10 season due to the effects of the H1N1 pandemic [40].

Table 5 gives mean values for $R_{S}$, the attack rate, and $S_{0}$ for influenza A across all seasons studied, with the exception of 2009 due to the H1N1 pandemic [38]. This table largely quantifies information in Figure 7 and Figure 8 and shows the relative change in value for seasons in which NPIs were in effect. We include the 22-23 season to highlight the substantial rebound in influenza A `post’-COVID, when NPIs have been largely abandoned. Instead of a return to the mean, we see a more severe influenza A year.

The pre-COVID mean values of $R_{S}$ and the attack rate are similar to those found in the literature [25,41,42,43].

## 4. Discussion

Our study largely confirms what was observed during the pandemic, that NPIs could have a substantial impact on other respiratory illnesses [1,2]. We have provided analysis that shows the impacts of large-scale behavioral and societal changes due to COVID-19 on influenza A. We have shown that while $R_{S}$ and the attack rate can be used to model NPIs, it is $S_{0}$ that is most consistently affected by behavioral and societal changes. We are cognizant that this may contradict the current practices of quantifying the effects of NPIs through β, but we believe this result can augment our understanding of how to model NPIs in the future. It is likely that the asymmetry of behaviors during a pandemic like COVID-19, may lead to population-level data being highly representative of a sub-population in a way that is not necessarily clear.

Complicating issues is the fact that every province has different testing protocols and the number of tests done varies year to year. The testing protocols are not publicly reported, and subjects for testing are generally not selected randomly from the population as a whole. Therefore, $S_{0}$ should be seen as an extension of the tested population. We account for the number of tests performed in each year but must assume that the testing protocols are largely consistent over our time period in order to make comparisons. The differences in the tested population account for the variance in our estimates of $S_{0}$ in all cases except for the years 2020–2022. Here, we see record low estimates of $S_{0}$ despite record high tests being performed. This suggests that we are broadly capturing the dynamics of NPIs during this time.

The results show that the effective initial susceptible population, $S_{0}$, is greatly affected by NPIs. The NPIs studied here, namely, masking and distancing, effectively reduce the pool of individuals to which the flu can spread. Decreases in $S_{0}$ are consistent across both years in which we are sure NPIs such as masking and social distancing are prevalent.

This is in contrast to the attack rate which increases in the 2021–2022 flu year. This year, unlike the year before it, sees a large-scale reopening of stores, restaurants and schools. The historically consistent attack rate coupled with the low estimate of $S_{0}$ suggests that there are sub-populations who were exposed to and spread influenza at pre-pandemic rates.

While $S_{0}$ and β are correlated in the expression for $R_{S}$, the additional information at hand, like the number of tests conducted and the cumulative number of infections in one season, gives us bounds on $S_{0}$ so that a model parameterized with the same $R_{S}$ but different $S_{0}$ will not yield the same fit.

We note that in Table 5, we see that by Spring 2022, the behavioral changes induced by the COVID-19 pandemic largely fall out of favor nationally, leading to a moderate rebound of influenza A. We see specifically in Ontario, where the data exist, that the reduction in $R_{S}$ and the attack rate are substantially lower than in the 20-21 season, and all parameters, including $S_{0}$, are trending back toward pre-pandemic levels.

Because they are inversely correlated, we see in our fitting that when $S_{0}$ sees a significant decrease, β, the contact rate, shows a significant increase. The fact that $R_{S}$ is lower during the 2020–2021 season shows that the increase in β is not exactly proportional to the decrease in $S_{0}$. Our interpretation is that there are effectively fewer individuals in the population, but they are generally those with higher contacts. This may inform estimates of effective contact rates of the subsets of the population who are considered ‘essential workers’ during a pandemic.

The data shown in Figure 3 show, particularly in the 2021–2022 season, that temporary regional lockdowns had minimal effect on influenza. This could be due to the timing of the lockdowns compared to the flu season of that year, or the relative length of flu season against the length and scope of lockdowns. This suggests that it was the social behaviors like masking and long-term policy and behavioral changes that drove a reduction in influenza A cases.

### 4.1. Limitations

With less biased and more randomized testing of influenza case data, this information could readily be applied to an entire population to monitor the effectiveness of NPIs during an outbreak of a novel infectious disease with similar transmission routes to the flu. As it stands, the raw parameters are subject to limitations of available data.

Due to these limitations, we focus on the relative change in epidemiological parameters of seasonal influenza A in each jurisdiction of study. In the 2020-2021 flu year, we see large changes (50% decrease) in the attack rate for influenza and about a 7% decrease in the reproduction number for influenza nation-wide, further suggesting that when forecasting and modeling disease transmission during periods of widespread societal changes, the effective population number is incredibly important and cannot be assumed to be the entire population. Using a known disease with similar routes of transmission may help guide estimates for effective population size during future outbreaks of novel infectious diseases.

The study also uses a very simple SIR model. With age-stratified data, a more robust model could be considered, which may lead to more accurate estimates for effective population size and the effective reproduction number.

### 4.2. Future Work

Future work includes expanding this framework and pipeline to other endemic, airborne pathogens in Canada to see if different pathogens are affected differently by the COVID-19 pandemic and associated behavioral changes. This work could help inform how certain policies will affect pathogens with differing properties. This could lead to targeted, effective NPIs being put in place once certain characteristics of infectious diseases are known.

We would also like to expand to different jurisdictions, perhaps all provinces and territories of Canada and other countries. Here, we focus on three large provincial jurisdictions to highlight that this fitting pipeline can be extended to the provincial level and produces consistent results.

Another curiosity is the increase in the reproduction number and the attack rate in the 2022-2023 season when NPIs and associated policies are phased out by the population at large. This may be tied to a possible decrease in uptake of the flu shot [44] (while this is a U.S.-based study, there is no academic study to our knowledge on the post-pandemic analysis of flu shot uptake in Canada), a reduction in cross-immunity through several seasons without exposure to influenza A, or cross-infection with COVID-19 [45]. Incorporating this into our multi-year study is the subject of future work.

## 5. Conclusions

Our study quantifies the efficacy of non-pharmaceutical interventions using a known airborne pathogen with a long history in Canada, and shows that NPIs are an effective tool against disease outbreak. We analyze the effects of COVID-19-era NPIs on influenza A across different jurisdictions in Canada. We see moderate changes in the seasonal reproductions number, $R_{S}$, for seasons which correspond to non-pharmaceutical interventions put in place for COVID-19. The 2020-2021 flu season saw the most dramatic reduction in $R_{S}$ across Canada with a 7.70% decrease. Interestingly, the following years saw an average 2.9% increase in reproduction number, indicating a reduction of NPIs and rebound. At a provincial level, most provinces saw continued decreases in $R_{S}$ until the 2022–2023 season when the reproduction number increased anywhere between 2% and 8.7%, depending on jurisdiction.

Our analysis shows that non-pharmaceutical interventions largely affect the effective population size of a jurisdiction, $S_{0}$, while having less of an effect on the contact rate. We see a two to three order of magnitude decrease in the effective population size for the years 2020 through 2022. This suggests that when considering NPIs, models should consider the fact that many individuals will self-isolate, effectively removing themselves from the population.

## Figures and Tables

**Table 1:** Vaccination rates for Canada from 2003 to 2023. Data sourced from Government of Canada reports [28,29,30].

Year	Vaccination Rate
2003	28
2005	34
2007	33
2009	30
2011	30
2013	31
2015	33
2016	32
2017	33
2018	32
2019	34
2020	39
2021	–
2022	34

**Table 2:** A comparison of the Akaike Information Criterion for the SIR model and SEIR model on the influenza A data. The comparison shows that the SIR model is favored, producing better fits with fewer parameters.

Province	Mean SIR AIC	Mean SEIR AIC
Ontario	330	378
Quebec	377	409
Alberta	−49	281
Canada	350	392

**Table 3:** Table of initial values for model fitting.

Parameter	Value	Fixed
β	0.000017	False
$S_{0}$	Total Seasonal Tests or Estimated	False
μ	7/2.5	True
I(0)	1	False
R(0)	0	True

**Table 4:** Fitted β, $S_{0}$, $R_{S}$ and computed attack rate for historical influenza A data for Canada, Ontario, Alberta, and Quebec. We can see from these data that the effects of NPIs put in place for COVID-19 have quantitative and qualitative effects on influenza A. We see that in the 2020–2021 season, a decrease in the seasonal reproduction number of influenza A is driven by decreases in the effective susceptible population. Note that Ontario experienced two waves of influenza A, one over 2021–2022 Winter, and one in Spring 2022. This secondary wave is denoted as 22 *.

	Canada				Alberta			
Season	beta	S0	R0	Attack Rate	beta	S0	R0	Attack Rate
99-00	0.00016622	20,462.8498	1.21473396	0.33483552	0.00151036	2457.57559	1.3256531	0.44755481
00-01	0.00084378	4028.24184	1.2139103	0.33065638	0.00541328	652.489663	1.26146686	0.3837101
01-02	0.00011566	27,349.6485	1.12971228	0.22064108	0.00135953	2456.36191	1.19267561	0.30468599
02-03	0.00030219	10,261.5702	1.10748155	0.18811571	0.0004846	5948.61466	1.02952665	0.05823518
03-04	6.05 × 10^−5^	52,142.6253	1.12735838	0.21760458	0.00100328	3427.25511	1.22803561	0.34724654
04-05	7.52 × 10^−5^	42,887.2548	1.15171921	0.25141863	0.0001697	17004.3483	1.03056289	0.06476137
05-06	0.00014047	22,397.7092	1.12365913	0.21172432	0.00101366	3147.40347	1.1394265	0.23432425
06-07	0.00010339	30,877.4116	1.140166	0.23537004	0.00037969	7983.6144	1.08259861	0.14970736
07-08	0.00010339	30,877.4116	1.140166	0.23537004	0.00072213	4368.71252	1.12670388	0.21618121
08-09	-	-	-	-	0.00072213	4368.71252	1.12670388	0.21618121
09-10	-	-	-	-	0.00043458	9780.91446	1.5180509	0.5943478
10-11	-	-	-	-	0.00058206	5376.39581	1.11764071	0.20299028
11-12	-	-	-	-	0.00047632	6596.29444	1.12213486	0.20949767
12-13	-	-	-	-	0.00041514	7979.79024	1.18311704	0.29272945
13-14	-	-	-	-	0.00037289	9565.34304	1.2738693	0.39670902
14-15	-	-	-	-	0.00037808	9393.82048	1.26842327	0.39104772
15-16	3.41 × 10^−5^	97,157.9315	1.18415282	0.294231	0.00023391	14,072.5729	1.17559655	0.28306968
16-17	-	-	-	-	0.00023164	14,074.295	1.16433378	0.26844891
17-18	1.82 × 10^−5^	172,721.58	1.11996118	0.20692799	0.00018046	18464.4047	1.19006223	0.30154145
18-19	1.09 × 10^−5^	28,0261.419	1.09060028	0.16248652	0.00010419	30140.5105	1.12155327	0.2100721
19-20	2.10 × 10^−5^	150,376.379	1.13046545	0.22182137	0.00014545	21,557.9891	1.11987677	0.20638974
20-21	0.01075928	275.094253	1.05707739	0.11709196	-	-	-	-
21-22	0.0035854	916.891558	1.17408102	0.284589	0.02212772	148.882271	1.17658036	0.28451531
22 *	6.93 × 10^−5^	48,541.2115	1.2010877	0.3249449	0.00028839	11209.0558	1.1544847	0.26096192
22-23	2.09 × 10^−5^	166,948.38	1.24519128	0.36630147	0.00015764	22485.5027	1.26594304	0.38850824
	Ontario				Quebec			
Season	beta	S0	R0	Attack Rate	beta	S0	R0	Attack Rate
99-00	0.00074962	4851.85059	1.29894834	0.4230064	0.00077977	4342.60522	1.20937034	0.32530217
00-01	0.00296546	1138.75531	1.20604749	0.32103014	0.00541212	649.274594	1.25498398	0.37677228
01-02	0.00088892	3725.36893	1.18270203	0.29216692	0.00064844	5494.58375	1.27246434	0.39525457
02-03	0.00177442	1942.89855	1.23125333	0.35153767	0.00153609	2133.80177	1.17061285	0.27659946
03-04	0.00046549	7789.40159	1.29495522	0.41827243	0.00039401	8571.04925	1.20610585	0.32109775
04-05	0.0003318	9905.84902	1.1738567	0.28081599	0.00031742	10,819.6541	1.22656879	0.34515459
05-06	0.00054947	5975.2032	1.17256237	0.27913414	0.00089931	3894.79047	1.25093969	0.37239322
06-07	0.00035166	9220.84317	1.15808148	0.25998643	0.00081906	4307.13253	1.25992366	0.38206704
07-08	0.00018104	16,750.107	1.08298577	0.14954702	0.00040096	8148.89306	1.16690804	0.27173346
08-09	9.53 × 10^−5^	32,397.7244	1.10266733	0.18025097	7.70 × 10^−5^	38,451.7123	1.05740624	0.10710804
09-10	-	-	-	-	-	-	-	-
10-11	0.00014013	23,577.9282	1.18001947	0.28877445	0.00015133	21651.0311	1.17014936	0.27602551
11-12	0.00086374	3730.03208	1.15063427	0.24987398	0.00050017	6561.96133	1.17216564	0.27861838
12-13	0.00014499	23,590.2503	1.22153541	0.33955597	0.00017254	20,963.9145	1.29181146	0.41490045
13-14	0.00022792	15,057.8952	1.22570189	0.34435907	0.00021373	16,006.0153	1.22177968	0.33962464
14-15	0.00011358	29,328.1298	1.18968194	0.30103562	0.00011507	29,182.9071	1.19935499	0.31292873
15-16	0.00015381	21,998.8842	1.2084727	0.32393718	0.0001359	25391.9529	1.23238512	0.35179066
16-17	9.34 × 10^−5^	34,930.4781	1.16542693	0.26989898	6.85 × 10^−5^	46505.5978	1.13827091	0.23275074
17-18	0.00011989	26,279.2055	1.12525571	0.21463281	5.66 × 10^−5^	57014.6818	1.15221044	0.25206623
18-19	0.0001122	27,927.841	1.11908254	0.2056516	5.07 × 10^−5^	62989.0653	1.14088207	0.23646914
19-20	0.0001419	23,132.3505	1.17228102	0.27940252	5.73 × 10^−5^	55742.2386	1.14034734	0.23568172
20-21	0.0093146	308.51246	1.02631054	0.06245293	-	-	-	-
21-22	0.01059945	311.293043	1.17840484	0.29000035	0.01365794	242.424461	1.18250705	0.29204132
22 *	0.00044435	7215.20374	1.14502913	0.26338778	0.00018216	19,384.4053	1.26108525	0.3836769
22-23	5.23 × 10^−5^	64,596.1557	1.20684163	0.32451261	7.90 × 10^−5^	45,305.5685	1.27857975	0.40156215

**Table 5:** Mean values of $R_{S}$ and attack rate of influenza A across all available pre-COVID seasons (with exception of the 2009–2010 season due to the H1N1 pandemic [38]) for Canada, Ontario, Alberta, and Quebec. We also report the percent change from the mean for the 20-21, 21-22, and 22-23 seasons. This secondary wave is denoted as 22 *.

	$R_{S}$				Attack Rate			
	Canada	Alberta	Ontario	Quebec	Canada	Alberta	Ontario	Quebec
Mean	1.15 ± 0.04	1.18 ± 0.11	1.18 ± 0.06	1.20 ± 0.06	0.24 ± 0.05	0.28 ± 0.13	0.29 ± 0.07	0.31 ± 0.07
% Change 20-21	−7.70	-	−13.29	-	−51.37	-	−78.40	-
% Change 21-22	2.51	−0.62	−0.45	−1.43	18.19	2.10	0.30	−5.45
% Change 22 *	4.87	−2.49	−3.27	5.12	34.95	−6.35	−8.90	24.22
% Change 22-23	8.72	6.93	1.96	6.57	52.13	39.42	12.24	30.01

## Data Availability

http://datadryad.org/share/NnAqoHExDbSSh3ZlRueaIRXc3ZhrkJF4Zb7VIfCQ_ic (accessed on 20 May 2025).
